# The crystal structures of three 3-methyl-1*H*-1,2,4-triazole-5-thio­nes, including a second polymorph of 4-[(*E*)-(5-bromo-2-hy­droxy­benzyl­idene)amino]-3-methyl-1*H*-1,2,4-triazole-5(4*H*)-thione and a redetermination of 4-amino-3-methyl-1*H*-1,2,4-triazole-5(4*H*)-thione

**DOI:** 10.1107/S205698901501422X

**Published:** 2015-08-06

**Authors:** Padmanabha S. Manjula, Balladka K. Sarojini, Hemmige S. Yathirajan, Mehmet Akkurt, Cem Cüneyt Ersanlı, Christopher Glidewell

**Affiliations:** a Department of Chemistry, P. A. College of Engineering, Nadupadavu, Montepadavu 574 153, Mangaluru, D. K., India; b Department of Industrial Chemistry, Mangalore University, Mangalagangothri 574 199, Mangaluru, D. K., India; c Department of Studies in Chemistry, University of Mysore, Manasagangotri, Mysore 570 006, India; d Department of Physics, Faculty of Sciences, Erciyes University, 38039 Kayseri, Turkey; e Department of Physics, Faculty of Arts and Sciences, Sinop University, 57010 Sinop, Turkey; f School of Chemistry, University of St Andrews, Fife KY16 9ST, Scotland

**Keywords:** crystal structures, 1*H*-1,2,4-triazole-5-thio­nes, polymorphism, hydrogen bonding

## Abstract

The structures of three 3-methyl-1*H*-1,2,4-triazole-5-thione derivatives are reported. The structure of 4-amino-3-methyl-1*H*-1,2,4-triazole-5(4*H*)-thione, C_3_H_6_N_4_S, (I), has been redetermined with an improved model for the H atoms: the non-H atoms of (I) all lie on mirror planes in space group *Pbcm*, and the H atoms of the methyl group are disordered over two sets of reflection-related atomic sites having occupancy 0.5: two independent N—H⋯S hydrogen bonds link the mol­ecules of compound (I) into complex sheets. The non-H atoms in the mol­ecules of 4-[(*E*)-(3,4-di­meth­oxy­benzyl­idene)amino]-3-methyl-1*H*-1,2,4-tri­azol-5(4*H*)-thione, C_12_H_14_N_4_O_2_S, (II), despite lying in general positions are close to planar, with a dihedral angle between the two rings of 6.31 (10)°: the mol­ecules of compound (II) are linked by a three-centre N—H⋯(O)_2_ hydrogen bond into a *C*(10)*C*(11)[*R*_1_^2^(5)] chain of rings. A second polymorph of 4-[(*E*)-(5-bromo-2-hy­droxy-5-bromo­benzyl­idene)amino]-3-methyl-1*H*-1,2,4-triazole-5(4*H*)-thione, C_10_H_9_BrN_4_OS, (III), has been identified; the non-H atoms are nearly co-planar with a dihedral angle between the two rings of 1.9 (4)°. There is an intra­molecular O—H⋯N hydrogen bond and the mol­ecules are linked by N—H⋯S hydrogen bonds, forming centrosymmetric *R*_2_^2^(8) dimers. Comparisons are made with some related structures.

## Abstract 2

The non-H atoms in the mol­ecules of three closely-related 4-amino-3-methyl-1*H*-1,2,4-triazole-5-thio­nes are either exactly or very nearly co-planar, and the compounds exhibit hydrogen-bonded supra­molecular assembly in two, one or zero dimensions.

## Chemical context   

**Figure 1:**
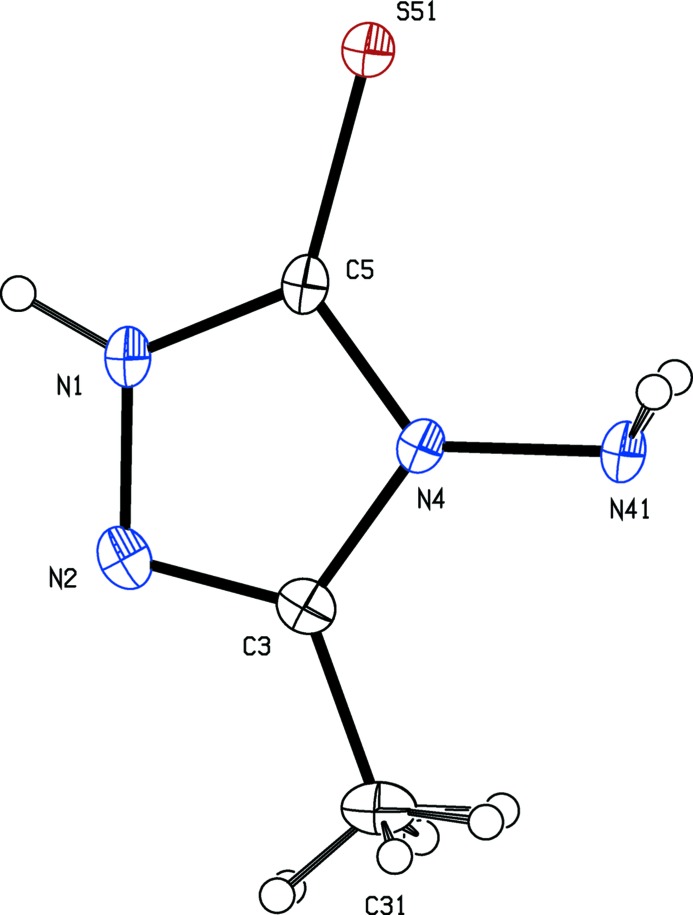
The mol­ecular structure of compound (I) showing the atom-labelling scheme. The non-H atoms all lie on a mirror plane and the H atom sites in the methyl group all have occupancy 0.5. Displacement ellipsoids are drawn at the 30% probability level.

**Figure 2:**
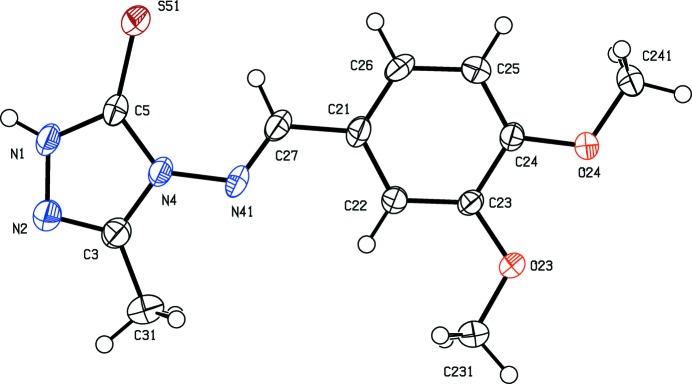
The mol­ecular structure of compound (II) showing the atom-labelling scheme. Displacement ellipsoids are drawn at the 30% probability level.

**Figure 3:**
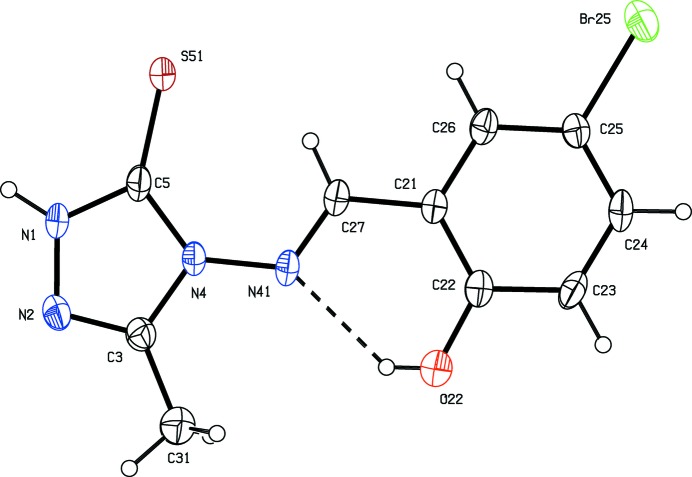
The mol­ecular structure of compound (III) in the monoclinic polymorph, showing the atom-labelling scheme and the intra­molecular O—H⋯N hydrogen bond. Displacement ellipsoids are drawn at the 30% probability level.

Heterocyclic compounds containing both nitro­gen and sulfur exhibit a wide variety of biological activities, including analgesic (Thieme *et al.*, 1973*a* ▸,*b* ▸), anti­hypertensive (Wei & Bell, 1981*a* ▸,*b* ▸), and anti-inflammatory activity (Dornow *et al.*, 1964 ▸), in addition to fungicidal (Malik *et al.*, 2011 ▸) and sedative action (Barrera *et al.*, 1985 ▸). Here we report the mol­ecular and crystal structures of three examples of 1,2,4-triazole-5-thio­nes, namely 4-amino-3-methyl-1*H*-1,2,4-triazole-5-thione, (I) (Fig. 1 ▸), 4-[(*E*)-(3,4-di­meth­oxy­benzyl­idene)amino]-3-methyl-1*H*-1,2,4-triazole-5-thione, (II) (Fig. 2 ▸), and 4-[(*E*)-(2-hy­droxy-5-bromo­benzyl­idene)amino]-3-methyl-1*H*-1,2,4-tri­azol-5-thione, (III) (Fig. 3 ▸).

The structure of compound (I) was briefly reported a number of years ago (Escobar-Valderrama *et al.*, 1989 ▸): however, there are some unexpected features in the reported structure, such as the implausibly wide range of the H—C—H angles in the methyl group, spanning the range 89–135°, and this report does not describe any supra­molecular inter­actions. A second report on this compound (Bigoli *et al.*, 1990 ▸) did not include H-atom coordinates, while in a third report (Sarala *et al.*, 2006 ▸) the structure was refined in space group *Pca*2_1_. However, a detailed examination of the atomic coordinates in this latter report using *PLATON* (Spek, 2009 ▸) found a 100% fit to space group *Pbcm*, indicating that an incorrect space group had probably been selected by these authors. Hence none of the previous reports on compound (I) can be regarded as satisfactory. Accordingly we have now taken the opportunity to re-determine the structure of compound (I) and to analyse in detail the effects of the hydrogen bonding. Compounds (II) and (III) were both prepared by condensation of compound (I) with the appropriate aryl aldehyde: crystallization of compound (III) from acetic acid yields a monoclinic polymorph in space group *P*2_1_/*c*, whereas crystallization from ethanol has been reported to provide a triclinic polymorph in space group *P* 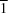 (Wang *et al.*, 2008 ▸). However, the unit-cell dimensions and the space group for (I) together confirm that the form of (I) studied here is the same as that in the original report, despite the use of different crystallization solvents, methanol here as opposed to ethanol in the original report.
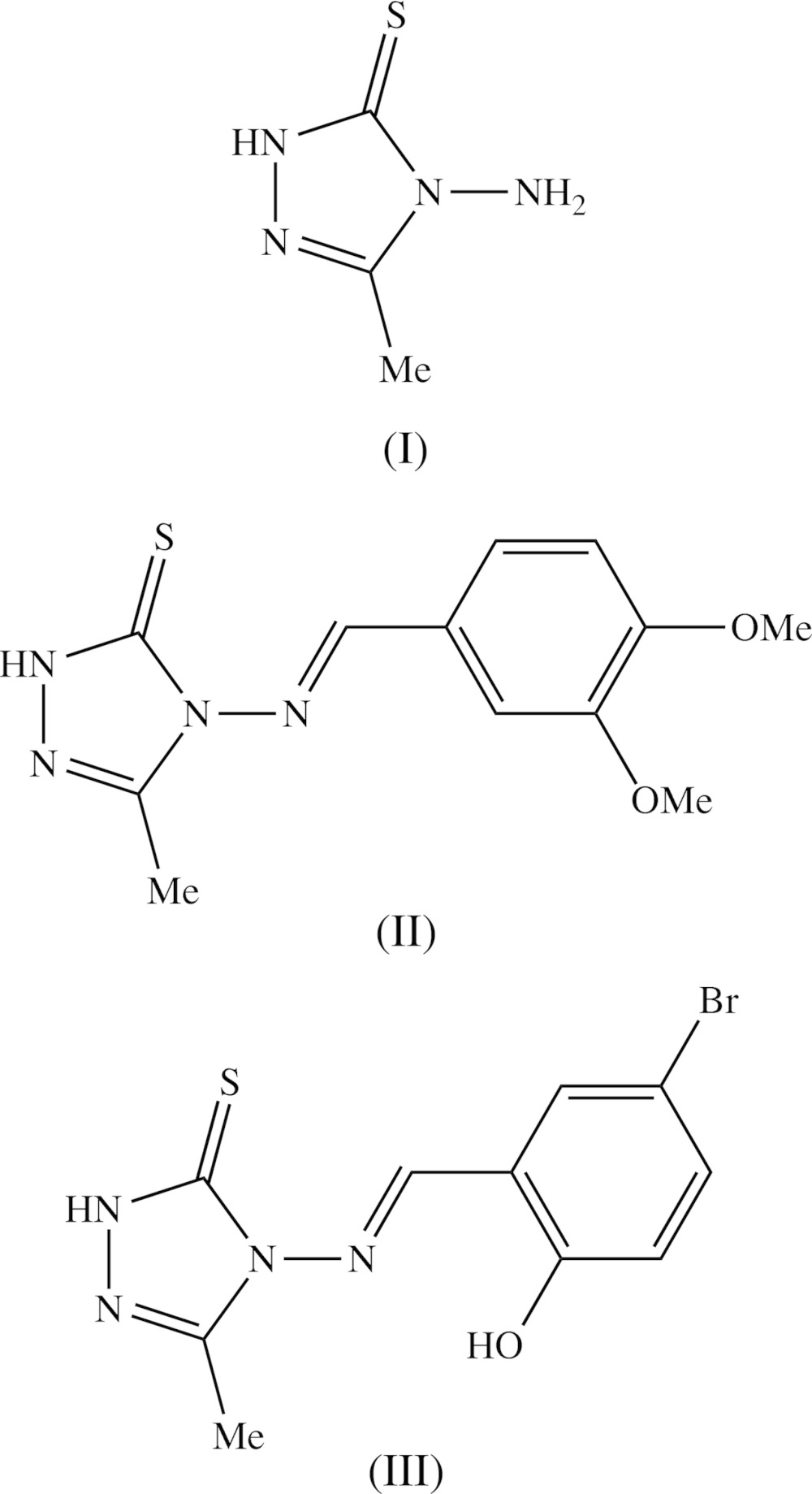


## Structural commentary   

Compound (I) crystallizes in the fairly uncommon ortho­rhom­bic space group *Pbcm*, which is represented by just 772 examples (about 0.06% of all entries) in the June 2015 release of the Cambridge Structural Database (Groom & Allen, 2014 ▸). All of the non-H atoms lie on a crystallographic mirror plane. The reference mol­ecule was selected as one lying on the plane at *z* = 1/4, and the orientation of the methyl group is such that the H atoms of this group are disordered over two sets of sites, all having occupancy 0.5 (Fig. 1 ▸). Although the mol­ecules of compounds (II) and (III) lie in general positions, the non-H atoms are close to co-planar in each case: an intra­molecular O—H⋯N hydrogen bond in (III) (Table 2) may contribute to this. Thus in compound (II) the dihedral angle between the two ring planes is 6.31 (10)° and, of the atoms in the mol­ecular skeleton, the maximum deviation from the mean plane of the skeletal atoms is 0.097 (2) Å for atom N41, with an r.m.s. deviation of 0.072 Å. In compound (III), the dihedral angle between the two ring planes is just 1.9 (4)°, and the maximum deviation of any atom from the mean plane of the mol­ecular skeleton is 0.038 (5) Å for atom C26, with an r.m.s deviation of 0.020 Å.

The meth­oxy C atoms in compound (II) are almost co-planar with the adjacent aryl ring, as indicated by the relevant torsional angles (Table 1 ▸), and the deviations of the two atoms from the plane of the aryl ring (C21–C26) are 0.017 (5) Å for atom C231 and 0.125 (5) Å for atom C241. Consistent with this, the pairs of exocyclic C—C—O angles at atoms C23 and C24 differ by *ca* 10°, as typically found when meth­oxy groups are co-planar with an aryl ring (Seip & Seip, 1973 ▸; Ferguson *et al.*, 1996 ▸). Corresponding bond distances within the triazole rings (Table 1 ▸) are very similar for all three compounds, as well as for the two polymorphs of compound (III): the values provide evidence for strong bond localization within the ring, with little or no hint of any aromatic-type delocalization, despite the presence of six π-electrons in rings of this type.

## Supra­molecular inter­actions   

**Figure 4:**
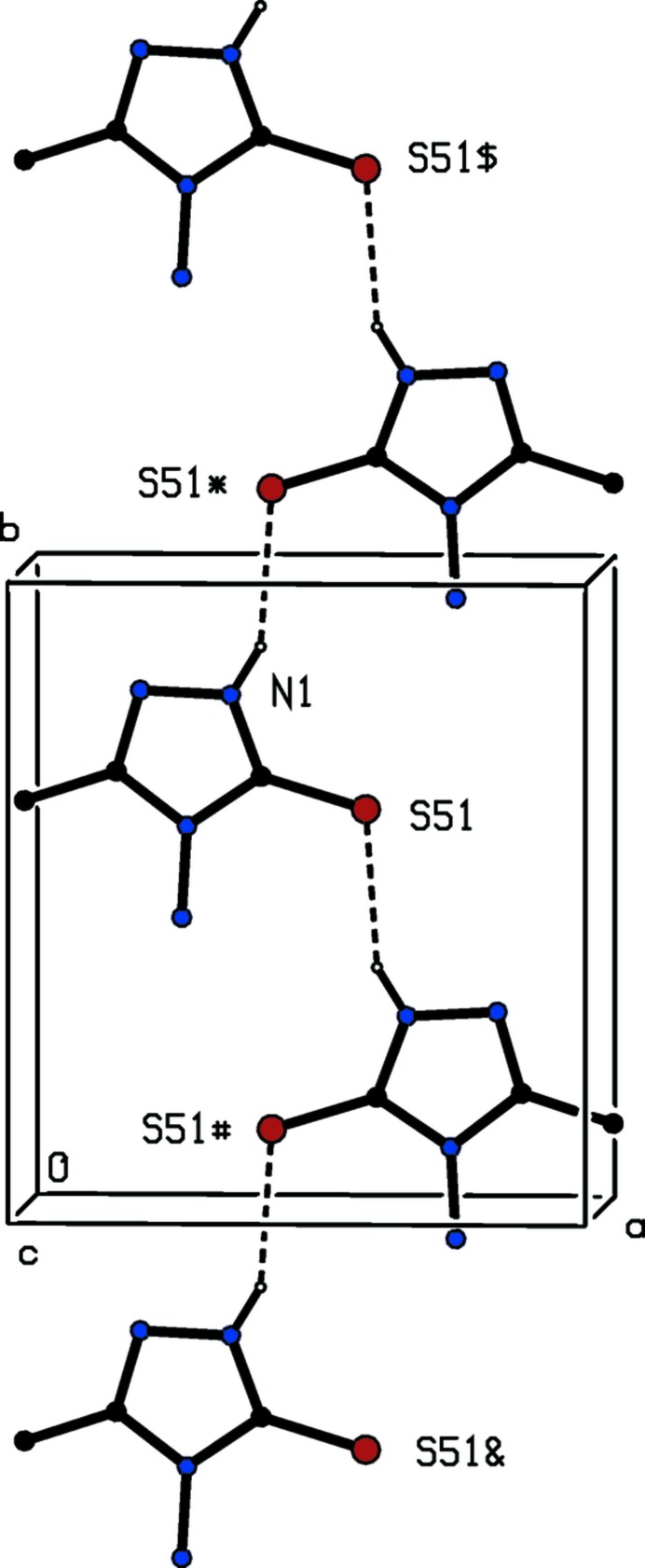
Part of the crystal structure of compound (I) showing the formation of a hydrogen-bonded *C*(4) chain running parallel to the [010] direction,. For the sake of clarity, the H atoms not involved in the motif shown have been omitted. The atoms marked with an asterisk (*), a hash (#), a dollar sign ($) or an ampersand (&) are at the symmetry positions (1 − *x*, 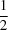 + *y*, 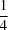), (1 − *x*, −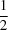 + *y*, 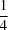), (*x*, 1 + *y*, 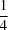) and (*x*, −1 + *y*, 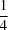) respectively.

**Figure 5:**
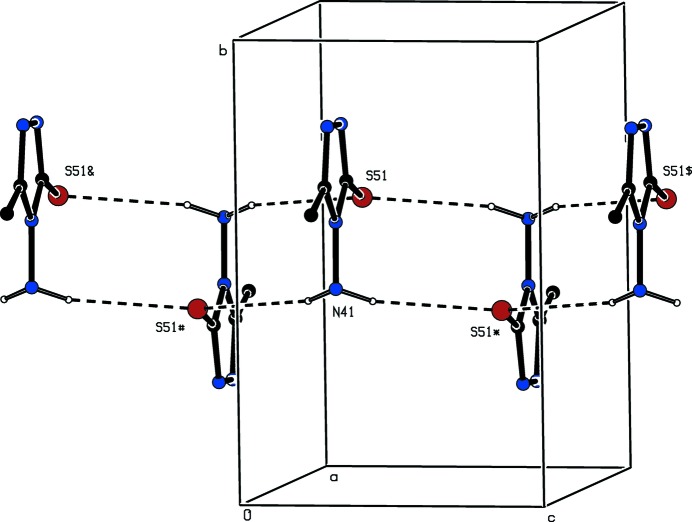
Part of the crystal structure of compound (I) showing the formation of hydrogen-bonded chain of edge-fused 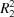
(10) rings running parallel to the [001] direction,. For the sake of clarity, the H atoms not involved in the motif shown have been omitted. The atoms marked with an asterisk (*), a hash (#), a dollar sign ($) or an ampersand (&) are at *z* = 0.75, *z* = −0.25, *z* = 1.25 and *z* = −0.75 respectively.

**Figure 6:**
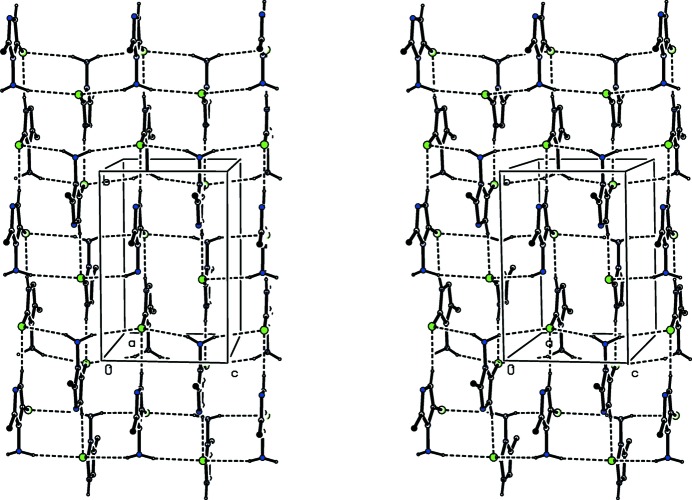
A stereoview of part of the crystal structure of compound (I) showing the formation of a hydrogen-bonded sheet lying parallel to (100). For the sake of clarity, the H atoms not involved in the motifs shown have been omitted.

In the crystal structure of compound (I) two independent hydrogen bonds (Table 2 ▸) of N—H⋯S type (Allen *et al.*, 1997 ▸) link the mol­ecules into complex sheets, whose formation is readily analysed in terms of two simple one-dimensional sub-structures (Ferguson *et al.*, 1998*a* ▸,*b* ▸; Gregson *et al.*, 2000 ▸). In the simpler of these two-sub-structures, mol­ecules related by the 2_1_screw axis along (1/2, *y*, 1/4) are linked by a hydrogen bond involving the ring N—H unit as the donor, forming a *C*(4) chain running parallel to the [010] direction (Fig. 4 ▸). The H atoms of the amino group also act as hydrogen-bond donors, and the effect is to link mol­ecules related by the 2_1_ screw axis along (1/2, 1/2, *z*) to form a chain of edge-fused 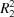
(10) rings running parallel to the [001] direction (Fig. 5 ▸). The combination of these two chain motifs, along [010] and [001] respectively, gives rise to a sheet lying parallel to (100) (Fig. 6 ▸): just one sheet of this type passes through each unit cell, but there are no direction-specific inter­actions between adjacent sheets. Hence the supra­moleuclar assembly of (I) is two dimensional.

**Figure 7:**
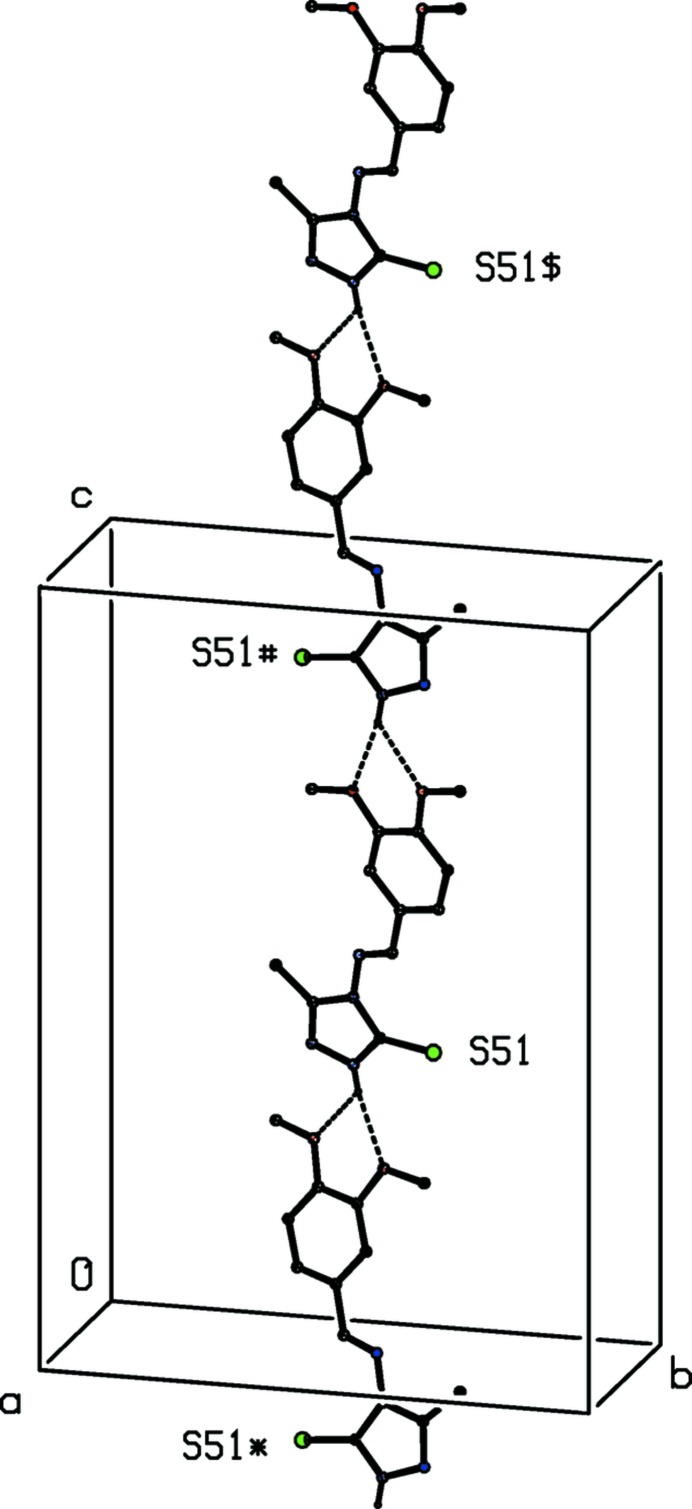
Part of the crystal structure of compound (II) showing the formation of a hydrogen-bonded *C*(10)*C*(11)[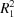
(5) chain of rings running parallel to the [001] direction. For the sake of clarity, the H atoms bonded to C atoms have been omitted. The atoms marked with an asterisk (*), a hash (#) or a dollar sign ($) are at the symmetry positions (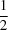 − *x*, 1 − *y*, −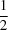 + *z*), (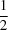 − *x*, 1 − *y*, 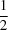 + *z*) and (*x*, *y*, 1 + *z*) respectively.

The N—H bond in compound (II) participates in the formation of a three-centre (bifurcated) N—H⋯(O,O) hydrogen-bond system, in which the two acceptors are the O atoms of the meth­oxy groups (Table 2 ▸): this three-centre system is markedly asymmetric, but it is planar within experimental uncertainty. The effect of this inter­action is to link mol­ecules related by the 2_1_ screw axis along (1/4, 1/2, *z*) to form a *C*(10)*C*(11)[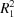
(5) chain of rings running parallel to the [001] direction (Fig. 7 ▸). Four chains of this type pass through each unit cell, but there are no direction-specific inter­actions between the chains: in particular, C—H⋯π(arene) hydrogen bonds and aromatic π–π stacking inter­actions are both absent from the crystal structure. Hence the supra­molecular assembly of (II) is one dimensional.

**Figure 8:**
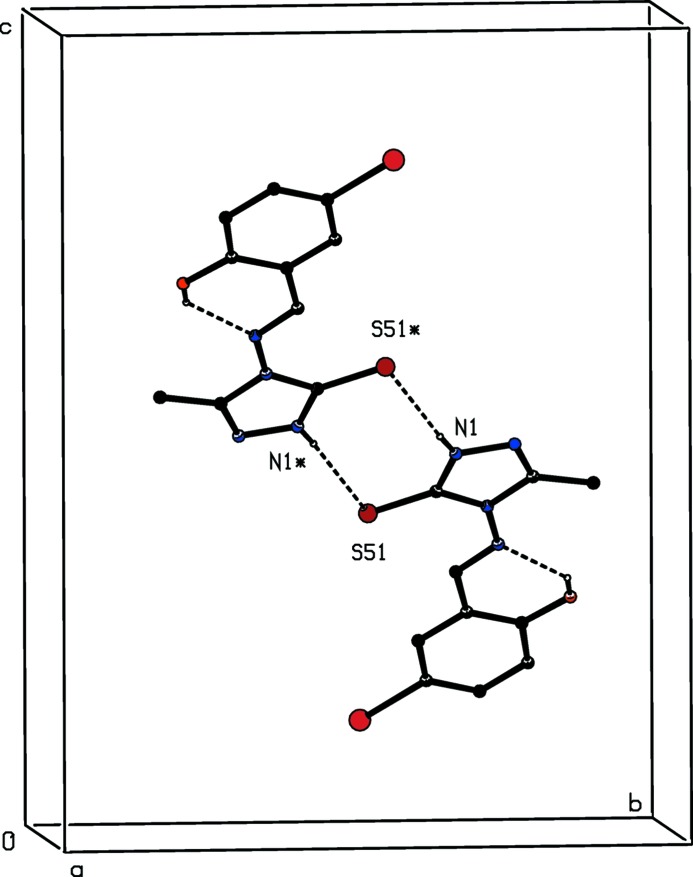
Part of the crystal structure of the monoclinic polymorph of compound (III) showing the formation of a hydrogen-bonded 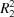
(8) dimer. For the sake of clarity, the H atoms bonded to C atoms have been omitted. The atoms marked with an asterisk are at the symmetry position (1 − *x*, 1 − *y*, 1 − *z*).

In addition to the intra­molecular hydrogen bond in the mol­ecule of compound (III), noted above, there is a single almost linear N—H⋯S hydrogen bond in this structure, which links inversion-related pairs of mol­ecules into a centrosymmetric dimer characterized by an 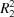
(8) motif (Fig. 8 ▸). There are no direction-specific inter­actions between adjacent dimers: as for compound (II), C—H⋯π(arene) hydrogen bonds and aromatic π–π stacking inter­actions are both absent from the crystal structure of compound (III). Hence the supra­molecular assembly in the monoclinic polymorph of (III) is finite or zero dimensional. The supra­molecular assembly in the triclinic polymorph was not analysed in the original report (Wang *et al.*, 2008 ▸). In fact, inversion-related pairs of mol­ecules are linked by N—H⋯S hydrogen bonds to form centrosymmetric 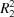
(8) dimers, exactly as in the monoclinic polymorph, but in the triclinic form these dimers are linked by an aromatic π–π stacking inter­action to form a π-stacked chain of hydrogen-bonded dimers running parallel to the [1
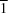
1] direction.

Thus for the three structures reported here, the supra­molecular assembly in compounds (I), (II) and the monoclinic polymorph of (III) is, respectively, two one and zero dimensional, while for the triclinic polymorph of (III) it is one dimensional.

## Database survey   

Here we briefly compare the supra­molecular assembly in compounds (IV)–(VIII) (see Scheme 2), which all have mol­ecular constitutions which are similar to those of compounds (II) and (III) reported here. 
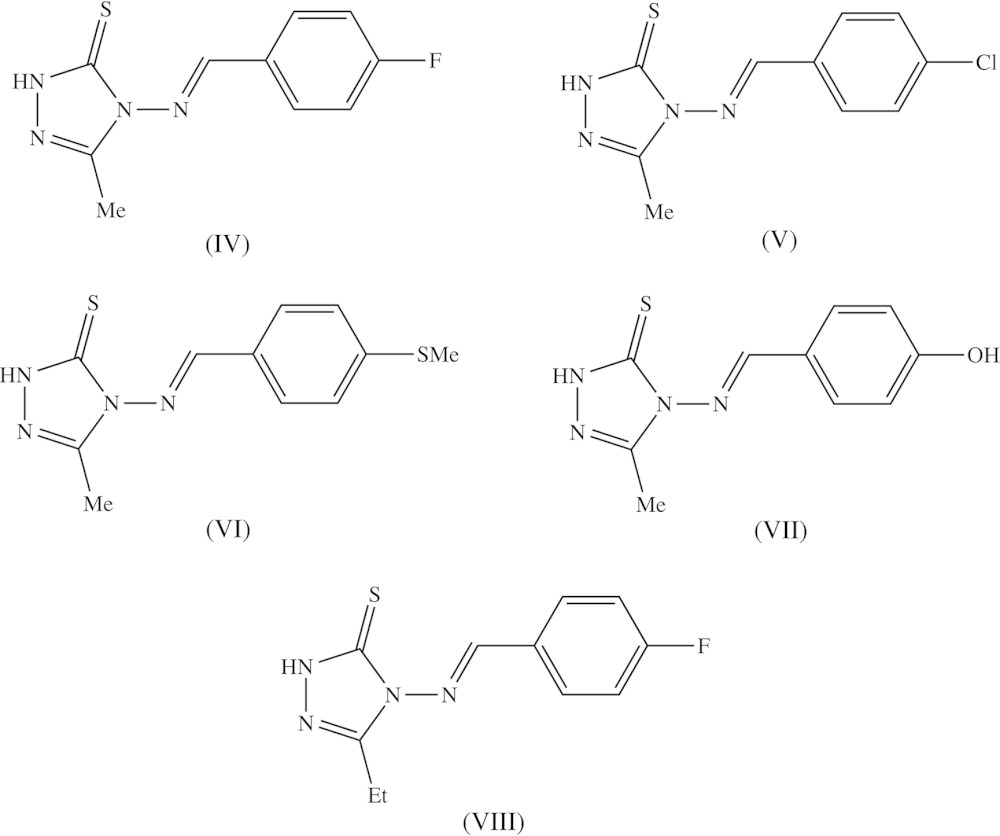


Compounds (IV) (Devarajegowda *et al.*, 2012 ▸) and (V) (Sarojini, Manjula, Kaur *et al.*, 2014 ▸) both crystallize in the triclinic space group *P* 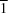, but they are not isostructural, as they crystallize with *Z*′ values of 2 and 1, respectively. However, their supra­molecular assembly is rather similar: in the structure of compound (IV), two independent N—H⋯S hydrogen bonds link the two mol­ecules of the selected asymmetric unit into a cyclic dimeric aggregate, while in compound (V) inversion-related pairs of mol­ecules are linked by N—H⋯S hydrogen bonds to form a cyclic centrosymmetric 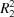
(8) dimer, analogous to those found in both polymorphs of compound (III). A similar centrosymmetric dimer is observed for compound (VI) (Sarojini *et al.*, 2013 ▸), but in compound (VII) (Sarojini, Manjula, Narayana *et al.*, 2014 ▸), motifs of this type form part of a ribbon containing alternating edge-fused 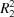
(8) and 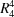
(28) rings running parallel to the [2
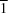
0] direction and in which both ring types are centrosymmetric. Finally, compound (VIII), which differs from (IV) in containing an ethyl substituent rather than a methyl substituent, but which crystallizes with *Z*′ = 1 in *P*2_1_/*c*. rather than with *Z*′ = 2 in *P* 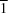 as for (IV), also contains a centrosymmetric 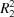
(8) dimeric aggregate (Jeyaseelan *et al.*, 2012 ▸).

## Synthesis and crystallization   

Colourless blocks of compound (I) were grown by slow evaporation, at ambient temperature and in the presence of air, of a solution in methanol. For the synthesis of compounds (II) and (III), to mixtures of 4-amino-3-methyl-1*H*-1,2,4-triazole-5(4*H*)-thione (0.01 mol) with either 3,4-di­meth­oxy­benzaldehyde (0.01 mol), for (II), or 5-bromo-2-hy­droxy­benzaldehyde (0.01 mol), for (III), in hot ethanol (15 ml) was added a catalytic qu­antity of concentrated sulfuric acid, and each mixture was then heated under reflux for 36 h. The mixtures were cooled to ambient temperature and the resulting solid products (II) and (III) were collected by filtration. For (II) and (III), colourless blocks were grown by slow evaporation, at ambient temperature and in the presence of air of solutions in either di­chloro­methane–methanol (1:1, *v*/*v*) for (II), or acetic acid for (III): m. p. (II) 471–473 K, (III) 465–467 K.

## Refinement   

Crystal data, data collection and structure refinement details are summarized in Table 3 ▸. All H atoms, including the disordered methyl H atoms in (I), were located in difference maps. The H atoms bonded to C atoms were then treated as riding atoms in geometrically idealized positions with C—H distances 0.93 Å (alkenyl and aromatic) or 0.96 Å (meth­yl) and with *U*_iso_(H) = k*U*_eq_(C), where k = 1.5 for the methyl groups, which were permitted to rotate but not to tilt, and 1.2 for all other H atoms bonded to C atoms. For the H atoms bonded to N atoms in compounds (I) and (II), the atomic coordinates were refined with *U*_iso_(H) = 1.2*U*_eq_(N), giving the N—H distances shown in Table 2 ▸. For compound (III), refinement of the atomic coordinates for the H atoms bonded to N and O atoms led to unacceptably large s.u.s of the resulting N—H and O—H distances: accordingly, these H atoms in (III) were permitted to ride on their carrier atoms with distances N—H = 0.86 Å and O—H = 0.82 Å, and with *U*_iso_(H) = 1.2*U*_eq_(N) or 1.5*U*_eq_(O). For each of compounds (II) and (III) the analysis of variance showed a large value of K for the very weak groups of reflections having *F*c/*F*c(max) in the range 0.000 < *F*c/*F*c(max) < 0.009 for (II) and 0.000 < *F*c/*F*c(max) < 0.015 for (III).

## Supplementary Material

Crystal structure: contains datablock(s) global, I, II, III. DOI: 10.1107/S205698901501422X/hb7466sup1.cif

Structure factors: contains datablock(s) I. DOI: 10.1107/S205698901501422X/hb7466Isup2.hkl

Structure factors: contains datablock(s) II. DOI: 10.1107/S205698901501422X/hb7466IIsup3.hkl

Structure factors: contains datablock(s) III. DOI: 10.1107/S205698901501422X/hb7466IIIsup4.hkl

Supporting information file. DOI: 10.1107/S205698901501422X/hb7466Isup5.cml

Supporting information file. DOI: 10.1107/S205698901501422X/hb7466IIsup6.cml

Supporting information file. DOI: 10.1107/S205698901501422X/hb7466IIIsup7.cml

CCDC references: 1415408, 1415407, 1415406

Additional supporting information:  crystallographic information; 3D view; checkCIF report

## Figures and Tables

**Table 1:** Selected geometric parameters (Å, °) for compounds (I)–(III)

Parameter	(I)	(II)	(III)	(III)
			*P*2_1_/*c*	*P* 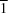
N1—N2	1.390 (2)	1.378 (3)	1.366 (7)	1.370 (5)
N2—C3	1.299 (3)	1.293 (3)	1.296 (7)	1.312 (5)
C3—N4	1.370 (3)	1.376 (3)	1.378 (7)	1.381 (5)
N4—C5	1.371 (2)	1.385 (3)	1.392 (7)	1.375 (5)
C5—N1	1.311 (2)	1.377 (3)	1.338 (7)	1.336 (5)
N4—N41	1.406 (2)	1.399 (3)	1.398 (7)	1.409 (5)
C5—S51	1.6833 (19)	1.675 (2)	1.644 (7)	1.681 (4)
N41—C27		1.261 (3)	1.279 (7)	1.285 (5)
				
N4—N41—C27		118.63 (19)	119.4 (5)	113.7 (3)
N41—C27—C21		121.6 (2)	119.0 (5)	120.0 (4)
C22—C23—O23		125.4 (2)		
C24—C23—O23		114.48 (19)		
C23—C24—O24		115.10 (19)		
C25—C24—O24		125.3 (2)		
				
N4—N41—C27—C21		−179.2 (2)	−179.2 (5)	176.5 (3)
N41—C27—C21—C22		4.9 (4)	0.5 (9)	−5.4 (6)
C22—C23—O23—C231		1.0 (4)		
C25—C24—O24—C241		−3.2 (4)		

Numerical data for the triclinic polymorph of compound (III) have been taken from the original report (Wang *et al.*, 2008), but the atom labels have been adjusted to match the systematic labels used for the structures reported here.

**Table 2:** Parameters (Å, °) for hydrogen bonds and short inter- and intra-mol­ecular contacts in compounds (I)–(III)

Compound	*D*—H⋯*A*		D—H	H⋯*A*	*D*⋯*A*	*D*—H⋯*A*
(I)	N1—H1⋯S51^i^		0.87 (3)	2.43 (3)	3.2326 (17)	153 (2)
	N41—H41⋯S51^ii^		0.882 (19)	2.753 (19)	3.5968 (8)	160.6 (16)
(II)	N1—H1⋯O23^iii^		0.81 (3)	2.29 (3)	3.075 (3)	166 (2)
	N1—H1⋯O24^iii^		0.81 (3)	2.41 (3)	2.978 (3)	128 (2)
(III)	N1—H1⋯S51^iv^		0.86	2.42	3.264 (6)	165
	O22—H22⋯N41		0.82	1.97	2.676 (6)	144

Symmetry codes: (i) 1 − *x*, 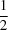 + *y*, 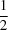 − *z*; (ii) 1 − *x*, 1 − *y*, −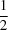 + *z*; (iii) 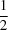 − *x*, 1 − *y*, −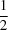 + *z*; (iv) 2 − *x*, 1 − *y*, 1 − *z*.

**Table 3:** Experimental details

	(I)	(II)	(III)
Crystal data			
Chemical formula	C_3_H_6_N_4_S	C_12_H_14_N_4_O_2_S	C_10_H_9_BrN_4_OS
*M*_r_	130.18	278.33	313.17
Crystal system, space group	Orthorhombic, *P* *b* *c* *m*	Orthorhombic, *P* *b* *c* *a*	Monoclinic, *P*2_1_/*c*
Temperature (K)	296	296	296
*a*, *b*, *c* (Å)	8.8682 (6), 9.8230 (6), 6.5427 (4)	7.3112 (4), 16.0793 (9), 22.8994 (13)	4.4122 (4), 14.7450 (13), 18.7911 (16)
α, β, γ (°)	90, 90, 90	90, 90, 90	90, 95.828 (3), 90
*V* (Å^3^)	569.95 (6)	2692.0 (3)	1216.19 (19)
*Z*	4	8	4
Radiation type	Mo *K*α	Mo *K*α	Mo *K*α
μ (mm^−1^)	0.46	0.24	3.54
Crystal size (mm)	0.24 × 0.18 × 0.15	0.21 × 0.15 × 0.11	0.22 × 0.19 × 0.15

Data collection			
Diffractometer	Bruker APEXII CCD	Bruker APEXII CCD	Bruker APEXII CCD
Absorption correction	Multi-scan (*SADABS*; Sheldrick, 2003 ▸)	Multi-scan (*SADABS*; Sheldrick, 2003 ▸)	Multi-scan (*SADABS*; Sheldrick, 2003 ▸)
*T*_min_, *T*_max_	0.876, 0.934	0.834, 0.974	0.376, 0.588
No. of measured, independent and observed [*I* > 2σ(*I*)] reflections	5602, 753, 687	26828, 3090, 2319	22155, 2270, 1913
*R*_int_	0.019	0.065	0.068
(sin θ/λ)_max_ (Å^−1^)	0.667	0.650	0.607

Refinement			
*R*[*F* ^2^ > 2σ(*F* ^2^)], *wR*(*F* ^2^), *S*	0.034, 0.086, 1.14	0.057, 0.123, 1.08	0.074, 0.131, 1.27
No. of reflections	753	3090	2270
No. of parameters	55	179	156
H-atom treatment	H atoms treated by a mixture of independent and constrained refinement	H atoms treated by a mixture of independent and constrained refinement	H-atom parameters constrained
Δρ_max_, Δρ_min_ (e Å^−3^)	0.33, −0.29	0.27, −0.24	0.60, −0.57

Computer programs: *APEX2* (Bruker, 2007 ▸), *SAINT* (Bruker, 2007 ▸), *SHELXS97* (Sheldrick, 2008 ▸), *SHELXL2014* (Sheldrick, 2015 ▸) and *PLATON* (Spek, 2009 ▸).

## supplementary crystallographic information

### (I) 4-Amino-3-methyl-1H-1,2,4-triazole-5(4H)-thione Crystal data

**Table:**

C_3_H_6_N_4_S	*D*_x_ = 1.517 Mg m^−^^3^
*M**_r_* = 130.18	Mo *K*α radiation, λ = 0.71073 Å
Orthorhombic, *P**b**c**m*	Cell parameters from 753 reflections
*a* = 8.8682 (6) Å	θ = 4.2–28.3°
*b* = 9.8230 (6) Å	µ = 0.46 mm^−^^1^
*c* = 6.5427 (4) Å	*T* = 296 K
*V* = 569.95 (6) Å^3^	Block, colourless
*Z* = 4	0.24 × 0.18 × 0.15 mm
*F*(000) = 272	

### (I) 4-Amino-3-methyl-1H-1,2,4-triazole-5(4H)-thione Data collection

**Table:**

Bruker APEXII CCD diffractometer	753 independent reflections
Radiation source: sealed tube	687 reflections with *I* > 2σ(*I*)
Graphite monochromator	*R*_int_ = 0.019
φ and ω scans	θ_max_ = 28.3°, θ_min_ = 4.2°
Absorption correction: multi-scan (SADABS; Sheldrick, 2003)	*h* = −11→11
*T*_min_ = 0.876, *T*_max_ = 0.934	*k* = −12→13
5602 measured reflections	*l* = −8→8

### (I) 4-Amino-3-methyl-1H-1,2,4-triazole-5(4H)-thione Refinement

**Table:**

Refinement on *F*^2^	0 restraints
Least-squares matrix: full	Hydrogen site location: mixed
*R*[*F*^2^ > 2σ(*F*^2^)] = 0.034	H atoms treated by a mixture of independent and constrained refinement
*wR*(*F*^2^) = 0.086	*w* = 1/[σ^2^(*F*_o_^2^) + (0.0324*P*)^2^ + 0.361*P*] where *P* = (*F*_o_^2^ + 2*F*_c_^2^)/3
*S* = 1.14	(Δ/σ)_max_ < 0.001
753 reflections	Δρ_max_ = 0.33 e Å^−^^3^
55 parameters	Δρ_min_ = −0.29 e Å^−^^3^

### (I) 4-Amino-3-methyl-1H-1,2,4-triazole-5(4H)-thione Special details

**Table:**

Geometry. All e.s.d.'s (except the e.s.d. in the dihedral angle between two l.s. planes) are estimated using the full covariance matrix. The cell e.s.d.'s are taken into account individually in the estimation of e.s.d.'s in distances, angles and torsion angles; correlations between e.s.d.'s in cell parameters are only used when they are defined by crystal symmetry. An approximate (isotropic) treatment of cell e.s.d.'s is used for estimating e.s.d.'s involving l.s. planes.

### (I) 4-Amino-3-methyl-1H-1,2,4-triazole-5(4H)-thione Fractional atomic coordinates and isotropic or equivalent isotropic displacement parameters (Å^2^)

**Table:**

	*x*	*y*	*z*	*U*_iso_*/*U*_eq_	Occ. (<1)
N1	0.34731 (19)	0.79125 (16)	0.2500	0.0287 (4)	
H1	0.399 (3)	0.867 (3)	0.2500	0.034*	
N2	0.1908 (2)	0.79840 (18)	0.2500	0.0344 (4)	
C3	0.1491 (2)	0.6716 (2)	0.2500	0.0292 (4)	
N4	0.27162 (18)	0.58666 (16)	0.2500	0.0240 (3)	
C5	0.4000 (2)	0.66435 (18)	0.2500	0.0230 (4)	
C31	−0.0098 (3)	0.6251 (3)	0.2500	0.0484 (7)	
H31A	−0.0407	0.6056	0.1125	0.073*	0.5
H31B	−0.0729	0.6952	0.3058	0.073*	0.5
H31C	−0.0187	0.5443	0.3317	0.073*	0.5
N41	0.2626 (2)	0.44381 (17)	0.2500	0.0324 (4)	
H41	0.314 (2)	0.4133 (18)	0.144 (3)	0.039*	
S51	0.58144 (5)	0.61399 (5)	0.2500	0.02978 (19)	

### (I) 4-Amino-3-methyl-1H-1,2,4-triazole-5(4H)-thione Atomic displacement parameters (Å^2^)

**Table:**

	*U*^11^	*U*^22^	*U*^33^	*U*^12^	*U*^13^	*U*^23^
N1	0.0302 (8)	0.0168 (7)	0.0392 (9)	−0.0006 (6)	0.000	0.000
N2	0.0326 (9)	0.0241 (8)	0.0464 (10)	0.0057 (7)	0.000	0.000
C3	0.0255 (9)	0.0285 (9)	0.0336 (10)	0.0028 (7)	0.000	0.000
N4	0.0237 (7)	0.0188 (7)	0.0295 (8)	−0.0031 (6)	0.000	0.000
C5	0.0287 (9)	0.0180 (8)	0.0222 (8)	−0.0023 (7)	0.000	0.000
C31	0.0225 (10)	0.0463 (13)	0.0763 (19)	0.0014 (9)	0.000	0.000
N41	0.0307 (9)	0.0162 (7)	0.0502 (11)	−0.0030 (6)	0.000	0.000
S51	0.0243 (3)	0.0224 (3)	0.0426 (3)	−0.00037 (16)	0.000	0.000

### (I) 4-Amino-3-methyl-1H-1,2,4-triazole-5(4H)-thione Geometric parameters (Å, º)

**Table:**

N1—C5	1.331 (2)	N4—N41	1.406 (2)
N1—N2	1.390 (2)	C5—S51	1.6833 (19)
N1—H1	0.88 (3)	C31—H31A	0.9600
N2—C3	1.299 (3)	C31—H31B	0.9600
C3—N4	1.370 (3)	C31—H31C	0.9600
C3—C31	1.481 (3)	N41—H41	0.883 (19)
N4—C5	1.371 (2)		
			
C5—N1—N2	113.45 (16)	N1—C5—N4	103.28 (16)
C5—N1—H1	128.0 (16)	N1—C5—S51	127.64 (15)
N2—N1—H1	118.6 (16)	N4—C5—S51	129.08 (14)
C3—N2—N1	103.63 (16)	C3—C31—H31A	109.5
N2—C3—N4	110.99 (17)	C3—C31—H31B	109.5
N2—C3—C31	124.50 (19)	H31A—C31—H31B	109.5
N4—C3—C31	124.51 (19)	C3—C31—H31C	109.5
C3—N4—C5	108.65 (17)	H31A—C31—H31C	109.5
C3—N4—N41	124.25 (16)	H31B—C31—H31C	109.5
C5—N4—N41	127.11 (16)	N4—N41—H41	108.0 (12)
			
C5—N1—N2—C3	0.0	N2—N1—C5—N4	0.0
N1—N2—C3—N4	0.0	N2—N1—C5—S51	180.0
N1—N2—C3—C31	180.0	C3—N4—C5—N1	0.0
N2—C3—N4—C5	0.0	N41—N4—C5—N1	180.0
C31—C3—N4—C5	180.0	C3—N4—C5—S51	180.0
N2—C3—N4—N41	180.0	N41—N4—C5—S51	0.0
C31—C3—N4—N41	0.0		

### (I) 4-Amino-3-methyl-1H-1,2,4-triazole-5(4H)-thione Hydrogen-bond geometry (Å, º)

**Table:**

*D*—H···*A*	*D*—H	H···*A*	*D*···*A*	*D*—H···*A*
N1—H1···S51^i^	0.87 (3)	2.43 (3)	3.2326 (17)	153 (2)
N41—H41···S51^ii^	0.882 (19)	2.753 (19)	3.5968 (8)	160.6 (16)

Symmetry codes: (i) −*x*+1, *y*+1/2, −*z*+1/2; (ii) −*x*+1, −*y*+1, *z*−1/2.

### (II) 4-[(E)-(3,4-Dimethoxybenzylidene)amino]-3-methyl-1H-1,2,4-triazole-5(4H)-thione Crystal data

**Table:**

C_12_H_14_N_4_O_2_S	*D*_x_ = 1.373 Mg m^−^^3^
*M**_r_* = 278.33	Mo *K*α radiation, λ = 0.71073 Å
Orthorhombic, *P**b**c**a*	Cell parameters from 3343 reflections
*a* = 7.3112 (4) Å	θ = 3.1–28.3°
*b* = 16.0793 (9) Å	µ = 0.24 mm^−^^1^
*c* = 22.8994 (13) Å	*T* = 296 K
*V* = 2692.0 (3) Å^3^	Block, colourless
*Z* = 8	0.21 × 0.15 × 0.11 mm
*F*(000) = 1168	

### (II) 4-[(E)-(3,4-Dimethoxybenzylidene)amino]-3-methyl-1H-1,2,4-triazole-5(4H)-thione Data collection

**Table:**

Bruker APEXII CCD diffractometer	3090 independent reflections
Radiation source: sealed tube	2319 reflections with *I* > 2σ(*I*)
Graphite monochromator	*R*_int_ = 0.065
φ and ω scans	θ_max_ = 27.5°, θ_min_ = 3.1°
Absorption correction: multi-scan (SADABS; Sheldrick, 2003)	*h* = −9→9
*T*_min_ = 0.834, *T*_max_ = 0.974	*k* = −20→20
26828 measured reflections	*l* = −29→29

### (II) 4-[(E)-(3,4-Dimethoxybenzylidene)amino]-3-methyl-1H-1,2,4-triazole-5(4H)-thione Refinement

**Table:**

Refinement on *F*^2^	Hydrogen site location: mixed
Least-squares matrix: full	H atoms treated by a mixture of independent and constrained refinement
*R*[*F*^2^ > 2σ(*F*^2^)] = 0.057	*w* = 1/[σ^2^(*F*_o_^2^) + (0.0344*P*)^2^ + 3.441*P*] where *P* = (*F*_o_^2^ + 2*F*_c_^2^)/3
*wR*(*F*^2^) = 0.123	(Δ/σ)_max_ = 0.001
*S* = 1.08	Δρ_max_ = 0.27 e Å^−^^3^
3090 reflections	Δρ_min_ = −0.24 e Å^−^^3^
179 parameters	Extinction correction: SHELXL2014 (Sheldrick, 2015), Fc^*^=kFc[1+0.001xFc^2^λ^3^/sin(2θ)]^-1/4^
0 restraints	Extinction coefficient: 0.0037 (8)

### (II) 4-[(E)-(3,4-Dimethoxybenzylidene)amino]-3-methyl-1H-1,2,4-triazole-5(4H)-thione Special details

**Table:**

Geometry. All e.s.d.'s (except the e.s.d. in the dihedral angle between two l.s. planes) are estimated using the full covariance matrix. The cell e.s.d.'s are taken into account individually in the estimation of e.s.d.'s in distances, angles and torsion angles; correlations between e.s.d.'s in cell parameters are only used when they are defined by crystal symmetry. An approximate (isotropic) treatment of cell e.s.d.'s is used for estimating e.s.d.'s involving l.s. planes.

### (II) 4-[(E)-(3,4-Dimethoxybenzylidene)amino]-3-methyl-1H-1,2,4-triazole-5(4H)-thione Fractional atomic coordinates and isotropic or equivalent isotropic displacement parameters (Å^2^)

**Table:**

	*x*	*y*	*z*	*U*_iso_*/*U*_eq_	
N1	0.1213 (3)	0.45675 (13)	0.33824 (9)	0.0329 (5)	
H1	0.115 (4)	0.4645 (17)	0.3034 (13)	0.040*	
N2	0.0910 (3)	0.37720 (13)	0.35845 (9)	0.0357 (5)	
C3	0.1225 (4)	0.38329 (15)	0.41387 (10)	0.0314 (5)	
N4	0.1725 (3)	0.46285 (12)	0.42911 (8)	0.0271 (5)	
C5	0.1697 (3)	0.51167 (15)	0.37928 (9)	0.0272 (5)	
C31	0.1081 (5)	0.31385 (16)	0.45639 (12)	0.0460 (7)	
H31A	0.2264	0.3027	0.4728	0.069*	
H31B	0.0640	0.2650	0.4368	0.069*	
H31C	0.0246	0.3289	0.4870	0.069*	
N41	0.2135 (3)	0.47940 (12)	0.48766 (8)	0.0293 (5)	
S51	0.20737 (12)	0.61356 (4)	0.36982 (3)	0.0438 (2)	
C27	0.2852 (4)	0.54836 (16)	0.50047 (10)	0.0341 (6)	
H27	0.3076	0.5867	0.4710	0.041*	
C21	0.3339 (3)	0.56941 (15)	0.56055 (9)	0.0281 (5)	
C22	0.3168 (3)	0.51237 (14)	0.60638 (9)	0.0253 (5)	
H22	0.2708	0.4594	0.5993	0.030*	
C23	0.3684 (3)	0.53506 (13)	0.66210 (9)	0.0238 (5)	
C24	0.4395 (3)	0.61528 (14)	0.67278 (9)	0.0250 (5)	
C25	0.4521 (4)	0.67139 (15)	0.62759 (11)	0.0335 (6)	
H25	0.4957	0.7248	0.6345	0.040*	
C26	0.3999 (4)	0.64807 (16)	0.57184 (10)	0.0359 (6)	
H26	0.4095	0.6862	0.5414	0.043*	
O23	0.3576 (3)	0.48540 (10)	0.71045 (7)	0.0357 (5)	
C231	0.2898 (5)	0.40291 (15)	0.70230 (11)	0.0450 (7)	
H23A	0.2829	0.3752	0.7394	0.067*	
H23B	0.3707	0.3728	0.6770	0.067*	
H23C	0.1702	0.4053	0.6851	0.067*	
O24	0.4902 (3)	0.63011 (10)	0.72903 (7)	0.0325 (4)	
C241	0.5711 (4)	0.70915 (15)	0.74105 (11)	0.0355 (6)	
H24A	0.4853	0.7525	0.7320	0.053*	
H24B	0.6789	0.7159	0.7176	0.053*	
H24C	0.6036	0.7122	0.7816	0.053*	

### (II) 4-[(E)-(3,4-Dimethoxybenzylidene)amino]-3-methyl-1H-1,2,4-triazole-5(4H)-thione Atomic displacement parameters (Å^2^)

**Table:**

	*U*^11^	*U*^22^	*U*^33^	*U*^12^	*U*^13^	*U*^23^
N1	0.0470 (14)	0.0347 (11)	0.0171 (10)	0.0033 (10)	−0.0027 (9)	0.0002 (9)
N2	0.0486 (14)	0.0319 (11)	0.0267 (11)	0.0008 (10)	−0.0023 (10)	−0.0006 (9)
C3	0.0360 (14)	0.0312 (12)	0.0271 (12)	0.0031 (11)	−0.0002 (11)	0.0017 (10)
N4	0.0337 (11)	0.0301 (10)	0.0174 (9)	0.0026 (9)	−0.0017 (8)	0.0012 (8)
C5	0.0292 (13)	0.0337 (12)	0.0186 (11)	0.0052 (10)	−0.0005 (9)	−0.0004 (9)
C31	0.062 (2)	0.0354 (14)	0.0401 (16)	−0.0047 (14)	−0.0008 (14)	0.0100 (12)
N41	0.0358 (12)	0.0375 (11)	0.0145 (9)	0.0030 (10)	−0.0031 (8)	0.0024 (8)
S51	0.0720 (5)	0.0312 (3)	0.0283 (3)	−0.0015 (3)	−0.0123 (3)	0.0053 (3)
C27	0.0417 (15)	0.0408 (14)	0.0198 (12)	−0.0043 (12)	−0.0022 (11)	0.0063 (10)
C21	0.0299 (13)	0.0352 (13)	0.0192 (11)	−0.0001 (11)	−0.0040 (9)	0.0021 (9)
C22	0.0293 (13)	0.0245 (11)	0.0221 (11)	0.0010 (10)	−0.0012 (9)	−0.0001 (9)
C23	0.0287 (12)	0.0231 (11)	0.0197 (11)	0.0019 (10)	0.0001 (9)	0.0030 (9)
C24	0.0267 (12)	0.0287 (11)	0.0194 (10)	−0.0009 (10)	−0.0009 (9)	−0.0004 (9)
C25	0.0411 (15)	0.0275 (12)	0.0319 (13)	−0.0085 (11)	−0.0048 (11)	0.0024 (10)
C26	0.0468 (16)	0.0370 (13)	0.0240 (12)	−0.0073 (12)	−0.0051 (11)	0.0132 (10)
O23	0.0599 (12)	0.0263 (8)	0.0210 (8)	−0.0093 (8)	−0.0061 (8)	0.0048 (6)
C231	0.073 (2)	0.0284 (13)	0.0334 (14)	−0.0118 (14)	−0.0106 (14)	0.0072 (11)
O24	0.0492 (11)	0.0271 (8)	0.0213 (8)	−0.0091 (8)	−0.0049 (8)	−0.0007 (6)
C241	0.0430 (15)	0.0343 (13)	0.0293 (14)	−0.0095 (12)	−0.0030 (11)	−0.0048 (10)

### (II) 4-[(E)-(3,4-Dimethoxybenzylidene)amino]-3-methyl-1H-1,2,4-triazole-5(4H)-thione Geometric parameters (Å, º)

**Table:**

N1—C5	1.337 (3)	C22—C23	1.380 (3)
N1—N2	1.378 (3)	C22—H22	0.9300
N1—H1	0.81 (3)	C23—O23	1.367 (3)
N2—C3	1.293 (3)	C23—C24	1.412 (3)
C3—N4	1.376 (3)	C24—O24	1.362 (3)
C3—C31	1.485 (3)	C24—C25	1.376 (3)
N4—C5	1.385 (3)	C25—C26	1.384 (3)
N4—N41	1.399 (3)	C25—H25	0.9300
C5—S51	1.675 (2)	C26—H26	0.9300
C31—H31A	0.9600	O23—C231	1.428 (3)
C31—H31B	0.9600	C231—H23A	0.9600
C31—H31C	0.9600	C231—H23B	0.9600
N41—C27	1.261 (3)	C231—H23C	0.9600
C27—C21	1.461 (3)	O24—C241	1.429 (3)
C27—H27	0.9300	C241—H24A	0.9600
C21—C26	1.378 (3)	C241—H24B	0.9600
C21—C22	1.399 (3)	C241—H24C	0.9600
			
C5—N1—N2	114.80 (19)	C21—C22—H22	120.1
C5—N1—H1	127 (2)	O23—C23—C22	125.4 (2)
N2—N1—H1	118 (2)	O23—C23—C24	114.48 (19)
C3—N2—N1	103.3 (2)	C22—C23—C24	120.2 (2)
N2—C3—N4	111.5 (2)	O24—C24—C25	125.3 (2)
N2—C3—C31	125.0 (2)	O24—C24—C23	115.10 (19)
N4—C3—C31	123.5 (2)	C25—C24—C23	119.6 (2)
C3—N4—C5	108.30 (19)	C24—C25—C26	119.8 (2)
C3—N4—N41	118.48 (18)	C24—C25—H25	120.1
C5—N4—N41	133.21 (19)	C26—C25—H25	120.1
N1—C5—N4	102.0 (2)	C21—C26—C25	121.2 (2)
N1—C5—S51	126.76 (18)	C21—C26—H26	119.4
N4—C5—S51	131.16 (18)	C25—C26—H26	119.4
C3—C31—H31A	109.5	C23—O23—C231	117.16 (18)
C3—C31—H31B	109.5	O23—C231—H23A	109.5
H31A—C31—H31B	109.5	O23—C231—H23B	109.5
C3—C31—H31C	109.5	H23A—C231—H23B	109.5
H31A—C31—H31C	109.5	O23—C231—H23C	109.5
H31B—C31—H31C	109.5	H23A—C231—H23C	109.5
C27—N41—N4	118.63 (19)	H23B—C231—H23C	109.5
N41—C27—C21	121.6 (2)	C24—O24—C241	116.80 (18)
N41—C27—H27	119.2	O24—C241—H24A	109.5
C21—C27—H27	119.2	O24—C241—H24B	109.5
C26—C21—C22	119.5 (2)	H24A—C241—H24B	109.5
C26—C21—C27	118.3 (2)	O24—C241—H24C	109.5
C22—C21—C27	122.2 (2)	H24A—C241—H24C	109.5
C23—C22—C21	119.7 (2)	H24B—C241—H24C	109.5
C23—C22—H22	120.1		
			
C5—N1—N2—C3	−0.2 (3)	C26—C21—C22—C23	−0.9 (4)
N1—N2—C3—N4	−0.5 (3)	C27—C21—C22—C23	178.7 (2)
N1—N2—C3—C31	179.8 (3)	C21—C22—C23—O23	179.7 (2)
N2—C3—N4—C5	1.0 (3)	C21—C22—C23—C24	−0.5 (4)
C31—C3—N4—C5	−179.2 (2)	O23—C23—C24—O24	1.5 (3)
N2—C3—N4—N41	−179.2 (2)	C22—C23—C24—O24	−178.4 (2)
C31—C3—N4—N41	0.5 (4)	O23—C23—C24—C25	−178.3 (2)
N2—N1—C5—N4	0.8 (3)	C22—C23—C24—C25	1.8 (4)
N2—N1—C5—S51	−177.20 (19)	O24—C24—C25—C26	178.4 (2)
C3—N4—C5—N1	−1.0 (3)	C23—C24—C25—C26	−1.8 (4)
N41—N4—C5—N1	179.2 (2)	C22—C21—C26—C25	0.9 (4)
C3—N4—C5—S51	176.8 (2)	C27—C21—C26—C25	−178.6 (3)
N41—N4—C5—S51	−2.9 (4)	C24—C25—C26—C21	0.4 (4)
C3—N4—N41—C27	169.6 (2)	C22—C23—O23—C231	1.0 (4)
C5—N4—N41—C27	−10.7 (4)	C24—C23—O23—C231	−178.9 (2)
N4—N41—C27—C21	−179.2 (2)	C25—C24—O24—C241	−3.2 (4)
N41—C27—C21—C26	−175.5 (3)	C23—C24—O24—C241	177.0 (2)
N41—C27—C21—C22	4.9 (4)		

### (II) 4-[(E)-(3,4-Dimethoxybenzylidene)amino]-3-methyl-1H-1,2,4-triazole-5(4H)-thione Hydrogen-bond geometry (Å, º)

**Table:**

*D*—H···*A*	*D*—H	H···*A*	*D*···*A*	*D*—H···*A*
N1—H1···O23^i^	0.81 (3)	2.29 (3)	3.075 (3)	166 (2)
N1—H1···O24^i^	0.81 (3)	2.41 (3)	2.978 (3)	128 (2)

Symmetry code: (i) −*x*+1/2, −*y*+1, *z*−1/2.

### (III) 4-[(E)-(5-Bromo-2-hydroxybenzylidene)amino]-3-methyl-1H-1,2,4-triazole-5(4H)-thione Crystal data

**Table:**

C_10_H_9_BrN_4_OS	*F*(000) = 624
*M**_r_* = 313.17	*D*_x_ = 1.710 Mg m^−^^3^
Monoclinic, *P*2_1_/*c*	Mo *K*α radiation, λ = 0.71073 Å
*a* = 4.4122 (4) Å	Cell parameters from 3002 reflections
*b* = 14.7450 (13) Å	θ = 3.5–28.3°
*c* = 18.7911 (16) Å	µ = 3.54 mm^−^^1^
β = 95.828 (3)°	*T* = 296 K
*V* = 1216.19 (19) Å^3^	Block, colourless
*Z* = 4	0.22 × 0.19 × 0.15 mm

### (III) 4-[(E)-(5-Bromo-2-hydroxybenzylidene)amino]-3-methyl-1H-1,2,4-triazole-5(4H)-thione Data collection

**Table:**

Bruker APEXII CCD diffractometer	2270 independent reflections
Radiation source: sealed tube	1913 reflections with *I* > 2σ(*I*)
Graphite monochromator	*R*_int_ = 0.068
φ and ω scans	θ_max_ = 25.6°, θ_min_ = 3.5°
Absorption correction: multi-scan (SADABS; Sheldrick, 2003)	*h* = −5→5
*T*_min_ = 0.376, *T*_max_ = 0.588	*k* = −17→17
22155 measured reflections	*l* = −22→22

### (III) 4-[(E)-(5-Bromo-2-hydroxybenzylidene)amino]-3-methyl-1H-1,2,4-triazole-5(4H)-thione Refinement

**Table:**

Refinement on *F*^2^	0 restraints
Least-squares matrix: full	Hydrogen site location: inferred from neighbouring sites
*R*[*F*^2^ > 2σ(*F*^2^)] = 0.074	H-atom parameters constrained
*wR*(*F*^2^) = 0.131	*w* = 1/[σ^2^(*F*_o_^2^) + 5.4069*P*] where *P* = (*F*_o_^2^ + 2*F*_c_^2^)/3
*S* = 1.27	(Δ/σ)_max_ < 0.001
2270 reflections	Δρ_max_ = 0.60 e Å^−^^3^
156 parameters	Δρ_min_ = −0.57 e Å^−^^3^

### (III) 4-[(E)-(5-Bromo-2-hydroxybenzylidene)amino]-3-methyl-1H-1,2,4-triazole-5(4H)-thione Special details

**Table:**

Geometry. All e.s.d.'s (except the e.s.d. in the dihedral angle between two l.s. planes) are estimated using the full covariance matrix. The cell e.s.d.'s are taken into account individually in the estimation of e.s.d.'s in distances, angles and torsion angles; correlations between e.s.d.'s in cell parameters are only used when they are defined by crystal symmetry. An approximate (isotropic) treatment of cell e.s.d.'s is used for estimating e.s.d.'s involving l.s. planes.

### (III) 4-[(E)-(5-Bromo-2-hydroxybenzylidene)amino]-3-methyl-1H-1,2,4-triazole-5(4H)-thione Fractional atomic coordinates and isotropic or equivalent isotropic displacement parameters (Å^2^)

**Table:**

	*x*	*y*	*z*	*U*_iso_*/*U*_eq_	
N1	0.9899 (12)	0.6271 (4)	0.4818 (3)	0.0498 (14)	
H1	1.0877	0.5851	0.5063	0.060*	
N2	1.0447 (12)	0.7171 (4)	0.4944 (3)	0.0463 (13)	
C3	0.8538 (14)	0.7579 (4)	0.4482 (3)	0.0402 (14)	
N4	0.6829 (10)	0.6958 (3)	0.4066 (2)	0.0353 (11)	
C5	0.7729 (14)	0.6089 (4)	0.4287 (3)	0.0431 (15)	
C31	0.8213 (17)	0.8564 (5)	0.4384 (4)	0.060 (2)	
H31A	0.6133	0.8736	0.4418	0.090*	
H31B	0.8783	0.8730	0.3922	0.090*	
H31C	0.9510	0.8871	0.4748	0.090*	
N41	0.4646 (10)	0.7266 (3)	0.3529 (2)	0.0357 (11)	
S51	0.6573 (5)	0.50707 (12)	0.39894 (11)	0.0689 (7)	
C27	0.3065 (13)	0.6689 (4)	0.3142 (3)	0.0388 (14)	
H27	0.3355	0.6071	0.3222	0.047*	
C21	0.0804 (11)	0.7002 (4)	0.2574 (3)	0.0308 (12)	
C22	0.0244 (14)	0.7913 (4)	0.2414 (3)	0.0408 (14)	
C23	−0.1947 (14)	0.8144 (5)	0.1853 (3)	0.0492 (17)	
H23	−0.2344	0.8751	0.1746	0.059*	
C24	−0.3526 (14)	0.7474 (5)	0.1456 (3)	0.0481 (16)	
H24	−0.4984	0.7628	0.1083	0.058*	
C25	−0.2931 (13)	0.6585 (4)	0.1616 (3)	0.0378 (14)	
C26	−0.0843 (13)	0.6330 (4)	0.2173 (3)	0.0385 (14)	
H26	−0.0526	0.5720	0.2282	0.046*	
O22	0.1721 (11)	0.8599 (3)	0.2776 (3)	0.0594 (13)	
H22	0.2946	0.8391	0.3091	0.089*	
Br25	−0.50755 (18)	0.56596 (6)	0.10681 (4)	0.0652 (3)	

### (III) 4-[(E)-(5-Bromo-2-hydroxybenzylidene)amino]-3-methyl-1H-1,2,4-triazole-5(4H)-thione Atomic displacement parameters (Å^2^)

**Table:**

	*U*^11^	*U*^22^	*U*^33^	*U*^12^	*U*^13^	*U*^23^
N1	0.055 (3)	0.045 (3)	0.044 (3)	0.004 (3)	−0.024 (3)	0.008 (2)
N2	0.049 (3)	0.050 (3)	0.037 (3)	0.003 (3)	−0.012 (2)	−0.005 (2)
C3	0.045 (4)	0.043 (4)	0.031 (3)	0.003 (3)	0.000 (3)	−0.001 (3)
N4	0.032 (3)	0.043 (3)	0.028 (2)	0.005 (2)	−0.006 (2)	0.003 (2)
C5	0.042 (3)	0.048 (4)	0.036 (3)	0.003 (3)	−0.011 (3)	0.008 (3)
C31	0.070 (5)	0.053 (4)	0.053 (4)	0.005 (4)	−0.008 (4)	0.000 (3)
N41	0.033 (3)	0.046 (3)	0.027 (2)	0.010 (2)	−0.003 (2)	0.005 (2)
S51	0.0795 (14)	0.0419 (10)	0.0740 (13)	−0.0012 (9)	−0.0482 (11)	0.0057 (9)
C27	0.035 (3)	0.047 (4)	0.033 (3)	0.009 (3)	−0.003 (3)	0.006 (3)
C21	0.025 (3)	0.042 (3)	0.026 (3)	0.004 (2)	0.005 (2)	0.006 (2)
C22	0.039 (3)	0.050 (4)	0.033 (3)	0.005 (3)	0.000 (3)	0.006 (3)
C23	0.051 (4)	0.046 (4)	0.048 (4)	0.012 (3)	−0.005 (3)	0.018 (3)
C24	0.043 (4)	0.065 (4)	0.034 (3)	0.011 (3)	−0.009 (3)	0.008 (3)
C25	0.037 (3)	0.046 (4)	0.030 (3)	0.000 (3)	−0.002 (2)	0.002 (3)
C26	0.039 (3)	0.047 (4)	0.029 (3)	0.010 (3)	0.003 (2)	0.004 (3)
O22	0.061 (3)	0.048 (3)	0.064 (3)	0.003 (2)	−0.018 (2)	0.005 (2)
Br25	0.0616 (5)	0.0720 (5)	0.0574 (4)	−0.0023 (4)	−0.0166 (3)	−0.0105 (4)

### (III) 4-[(E)-(5-Bromo-2-hydroxybenzylidene)amino]-3-methyl-1H-1,2,4-triazole-5(4H)-thione Geometric parameters (Å, º)

**Table:**

N1—C5	1.338 (7)	C27—H27	0.9300
N1—N2	1.366 (7)	C21—C22	1.393 (8)
N1—H1	0.8600	C21—C26	1.402 (8)
N2—C3	1.296 (7)	C22—O22	1.349 (7)
C3—N4	1.378 (7)	C22—C23	1.399 (8)
C3—C31	1.470 (9)	C23—C24	1.383 (9)
N4—C5	1.392 (7)	C23—H23	0.9300
N4—N41	1.398 (6)	C24—C25	1.364 (9)
C5—S51	1.664 (7)	C24—H24	0.9300
C31—H31A	0.9600	C25—C26	1.374 (7)
C31—H31B	0.9600	C25—Br25	1.903 (6)
C31—H31C	0.9600	C26—H26	0.9300
N41—C27	1.279 (7)	O22—H22	0.8200
C27—C21	1.461 (7)		
			
C5—N1—N2	115.1 (5)	N41—C27—H27	120.1
C5—N1—H1	122.5	C21—C27—H27	120.1
N2—N1—H1	122.4	C22—C21—C26	119.7 (5)
C3—N2—N1	104.1 (5)	C22—C21—C27	123.8 (5)
N2—C3—N4	110.7 (5)	C26—C21—C27	116.6 (5)
N2—C3—C31	126.3 (6)	O22—C22—C21	123.3 (5)
N4—C3—C31	123.1 (5)	O22—C22—C23	117.3 (6)
C3—N4—C5	108.6 (4)	C21—C22—C23	119.4 (6)
C3—N4—N41	119.4 (5)	C24—C23—C22	120.3 (6)
C5—N4—N41	132.0 (5)	C24—C23—H23	119.9
N1—C5—N4	101.5 (5)	C22—C23—H23	119.9
N1—C5—S51	127.0 (5)	C25—C24—C23	119.5 (5)
N4—C5—S51	131.4 (4)	C25—C24—H24	120.2
C3—C31—H31A	109.5	C23—C24—H24	120.2
C3—C31—H31B	109.5	C24—C25—C26	122.0 (6)
H31A—C31—H31B	109.5	C24—C25—Br25	119.7 (4)
C3—C31—H31C	109.5	C26—C25—Br25	118.3 (5)
H31A—C31—H31C	109.5	C25—C26—C21	119.1 (6)
H31B—C31—H31C	109.5	C25—C26—H26	120.4
C27—N41—N4	119.4 (5)	C21—C26—H26	120.4
N41—C27—C21	119.9 (5)	C22—O22—H22	109.5
			
C5—N1—N2—C3	0.6 (8)	N41—C27—C21—C22	0.5 (9)
N1—N2—C3—N4	−0.5 (7)	N41—C27—C21—C26	−179.9 (5)
N1—N2—C3—C31	−179.1 (7)	C26—C21—C22—O22	179.7 (6)
N2—C3—N4—C5	0.3 (7)	C27—C21—C22—O22	−0.7 (9)
C31—C3—N4—C5	178.9 (6)	C26—C21—C22—C23	−0.4 (9)
N2—C3—N4—N41	−179.6 (5)	C27—C21—C22—C23	179.2 (6)
C31—C3—N4—N41	−0.9 (9)	O22—C22—C23—C24	179.5 (6)
N2—N1—C5—N4	−0.5 (7)	C21—C22—C23—C24	−0.5 (10)
N2—N1—C5—S51	178.6 (5)	C22—C23—C24—C25	0.0 (10)
C3—N4—C5—N1	0.1 (7)	C23—C24—C25—C26	1.4 (10)
N41—N4—C5—N1	179.9 (6)	C23—C24—C25—Br25	−179.5 (5)
C3—N4—C5—S51	−178.9 (6)	C24—C25—C26—C21	−2.3 (9)
N41—N4—C5—S51	0.9 (11)	Br25—C25—C26—C21	178.7 (4)
C3—N4—N41—C27	179.8 (5)	C22—C21—C26—C25	1.7 (8)
C5—N4—N41—C27	0.1 (9)	C27—C21—C26—C25	−177.9 (5)
N4—N41—C27—C21	−179.2 (5)		

### (III) 4-[(E)-(5-Bromo-2-hydroxybenzylidene)amino]-3-methyl-1H-1,2,4-triazole-5(4H)-thione Hydrogen-bond geometry (Å, º)

**Table:**

*D*—H···*A*	*D*—H	H···*A*	*D*···*A*	*D*—H···*A*
N1—H1···S51^i^	0.86	2.42	3.264 (6)	165
O22—H22···N41	0.82	1.97	2.676 (6)	144

Symmetry code: (i) −*x*+2, −*y*+1, −*z*+1.
